# Study protocol for a non-inferiority trial of a blended smoking cessation treatment versus face-to-face treatment (LiveSmokefree-Study)

**DOI:** 10.1186/s12889-016-3851-x

**Published:** 2016-11-24

**Authors:** Lutz Siemer, Marcel E. Pieterse, Marjolein G. J. Brusse-Keizer, Marloes G. Postel, Somaya Ben Allouch, Robbert Sanderman

**Affiliations:** 1 Research Group Technology, Health & Care, Saxion University of Applied Sciences, Postbus 70.000, 7500KB Enschede, The Netherlands; 2 Centre for eHealth and Well-being Research, University of Twente, Enschede, The Netherlands; 3 Medisch Spectrum Twente, Enschede, The Netherlands; 4 Tactus, Enschede, The Netherlands

**Keywords:** Health care sector, Tobacco use disorder, Smoking cessation, Randomized controlled trial, Internet-based-treatment, Blended care, Blended treatment, Online counseling, eHealth

## Abstract

**Background:**

Smoking cessation can significantly reduce the risk of developing smoking-related diseases. Several face-to-face and web-based treatments have shown to be effective. Blending of web-based and face-to-face treatment is expected to improve smoking cessation treatment. The primary objective of this study is to compare the prolonged abstinence rate of the blended smoking cessation treatment with the face-to-face treatment. Secondary objectives are to assess the benefits of blended treatment in terms of cost effectiveness and patient satisfaction, and to identify mechanisms underlying successful smoking cessation.

**Methods/Design:**

This study will be a single-center randomized controlled non-inferiority-trial with parallel group design. Patients (*n* = 344) will be randomly assigned to either the blended or the face-to-face group. Both treatments will consist of ten sessions with equal content held within 6 months. In the blended treatment five out of ten sessions will be delivered online. The treatments will cover the majority of behavior change techniques that are evidence-based within smoking cessation counseling. All face-to-face sessions in both treatments will take place at the outpatient smoking cessation clinic of a hospital. The primary outcome parameter will be biochemically validated prolonged abstinence at 15 months from the start of the smoking cessation treatment.

**Discussion:**

This RCT will be the first study to examine the effectiveness of a blended smoking cessation treatment. It will also be the first study to explore patient satisfaction, adherence, cost-effectiveness, and the clinically relevant influencing factors of a blended smoking cessation treatment. The findings of this RCT are expected to substantially strengthen the base of evidence available to inform the development and delivery of smoking cessation treatment.

**Trial registration:**

Nederlands Trialregister NTR5113. Registered 24 March 2015.

## Background

Killing nearly six million people a year, smoking tobacco is one of the biggest public health threats. Of the smokers who are aware of the dangers of tobacco the majority want to quit [1]. Although a proportion of smokers quit without professional support [2], counseling and medication can more than double the success rate [1]. Success rates of smoking cessation treatments (5-months-after-treatment) range between 8,5% (minimal or no counseling or self-help) and 27,6% (intense counseling & medication) [3], depending on (1) contact time and intensity, (2) number and length of sessions, (3) number and type of clinicians involved, and (4) number and type of counselling formats and interventions. A treatment comparable to the ones of this trial has shown to lead to a cotinine-validated prolonged 12 months’ abstinence rate of 10% (based on intention-to-treat analysis) [4, 5].

Traditionally, smoking cessation treatment is offered as face-to-face counseling. With the rise of the internet, web-based treatment offers an additional channel for effective smoking cessation [6]. Nowadays face-to-face treatment and web-based treatment are usually offered separately. An integration of web-based and face-to-face treatments (blended treatment) is expected to combine the “best of both worlds” [7] as this will allow the strengths of one to offset the weaknesses inherent in the other [8].

The weaknesses of face-to-face treatment that can be offset by the strengths of web-based treatment refer to (1) therapist drift; (2) patients’ no-show and (3) travel costs. (1) Face-to-face treatments often suffer from therapist drift [9]. This drift can be reduced by the protocolled nature of web-based treatments, which have shown to lead to higher treatment integrity [10]. (2) Patients’ no-shows result in time lost both for the counselors and the patients. In BSCT counselors can replace patients not showing up with online work, which can be planned flexibly as the process of online communication with the patients occurs asynchronously. Patients that miss a face-to-face session can still access their personal online dossier and continue treatment autonomously (e.g. psycho-education, exercises, and summaries of counseling conversations). As both counselors and patients can use their time more efficiently this can result in offering treatment to more patients [11]. (3) Travelling to the smoking cessation clinic to attend a face-to-face meeting with the counselors is both time consuming and costly for the client. Web-based sessions do not require showing up at the clinic during the normal business hours, because they can be done e.g. at home in the evening. This reduces work time lost as well as travel related costs for the patients [9, 12].

The weaknesses of web-based treatment that can be offset by the strengths of face-to-face treatment refer to (1) poor engagement of patients and (2) tailoring of interventions. (1) A common problem of web-based treatment is poor engagement of users due to the small amount of personal contact [13]. Face-to-face treatment offers more personal contact and may therefor result in a higher commitment of the patients. (2) Web-based treatments are supposed to offer more tailoring [6]. Face-to-face treatment can offer greater flexibility in customizing interventions to the patients’ needs by for example explaining therapeutic interventions or providing additional information for diagnostic purpose or case management [9, 14, 15].

To the best of our knowledge there has been no research on the potential benefits of a blended treatment for smoking cessation. The primary objective of this research is to find out if a blended smoking cessation treatment (BSCT) results in non-inferior quit rates compared to a face-to-face treatment as usual (TAU). Secondary objectives are to assess whether (1) patients are more satisfied with BSCT; (2) BSCT reduces treatment costs; (3) there is link between quitting and adherence in BSCT; (4) there are moderators or mediators predicting treatment outcome; and (5) how the treatment can further be improved.

## Methods/Design

The study will be a single-centre randomized controlled non-inferiority trial with a parallel group design.

### Study population

The study population will consist of the patients of the outpatient smoking cessation clinic at Medisch Spectrum Twente (MST), Enschede, The Netherlands. These patients will be referred to the clinic by treating physicians of the hospital or by their GPs.

### Inclusion/Exclusion criteria

Participants of this study will be smokers who admit themselves to the cessation clinic (indicating readiness to quit), who are at least 18 years old, who are currently daily smokers (at least one cigarette/day), and who are able to both access websites and communicate by email (both verified during intake procedure by asking whether the participant has internet access at home, and a current email address). People who are not able to read or write in the Dutch language will be excluded from this study.

### Recruitment

Participant will be recruited from those patients that have signed up for smoking cessation treatment at the outpatient smoking cessation clinic. Based on earlier studies and the criteria for participation, we expect that a majority of patients will be eligible for this study. Participation in the study will be voluntary and patients will not receive any payment for participation.

### Randomization

Patients will be randomly assigned to either BSCT or TAU. Randomization will be performed at the individual level (allocation ratio 1:1) using QMinim Online Minimization (http://qminim.sourceforge.net/) [16]. The minimization will be stratified according to: (1) level of internet skills [17]; (2) level of nicotine dependence [18]); and (3) the quitting strategy favored by the patient (stop at once, gradual change, scheduled reduced smoking; for details see below the description of the study intervention). The data used for the QMinim minimization will be collected using the baseline questionnaire, which will be filled in online by the patient at home.

### Blinding

The study will be an open label study, as it is self-evidently impossible (due to the nature of the treatment conditions) to blind the staff and patients that are involved.

### Study intervention

BSCT will be a combination of face-to-face treatment combined with web-based treatment into one integrated smoking cessation treatment that can be delivered in conventional smoking cessation clinics [12, 14]. Both BSCT and TAU will be provided by the Outpatient Smoking Cessation Clinic (SRP), which is part of the Department of Pulmonary Medicine of MST hospital. The web-based interaction of BSCT – which patients will do e.g. at home - will make use of Tactus Addiction Treatment’s website http://www.rokendebaas.nl. Out of this web-based treatment five session have been adopted for the integration in BSCT (Table 1). The SRP team consists of a pulmonologist and three qualified stop-smoking counsellors. The counsellors are registered on the Dutch Quality Register of qualified smoking cessation counsellors (http://www.kwaliteitsregisterstopmetroken.nl). Both treatments fulfil the requirements of the Dutch care module for smoking cessation [19] which is derived from the evidence-based Dutch Guideline Treatment Tobacco Addiction [20]. The costs of smoking cessation treatment will be reimbursed by the patient’s health care insurance.

**Table 1:** Distribution of the behaviour change techniques in the face-to-face and online session in BSCT and TAU

Session/week	Name (Code) of the main behavioural change techniques according to [20]	TAU	BSCT
Session 1, week 1 Goal setting	Provide information on consequences of smoking and smoking cessation (BM1) Provide rewards contingent on successfully stopping smoking (BM4) Identify reasons for wanting and not wanting to stop smoking (BM9) Facilitate goal setting (BS4) Prompt self-recording (BS6) Advise on stop-smoking medication (A1) Advise on/facilitate use of social support (A2) Build general rapport (RC1) Explain expectations regarding treatment programme (RC4)	Face-to-face	Face-to-face
Session 2, week 3 Measures for self-control	Provide feedback on current behaviour (BM3) Provide rewards contingent on effort or progress (BM7) Facilitate barrier identification and problem solving (BS1) Facilitate relapse prevention and coping (BS2) Prompt review of goals (BS5) Prompt self-recording (BS6) Advise on changing routine (BS7) Tailor interactions appropriately (RD1)	Face-to-face	Online
Session 3, week 5 Dealing with withdrawal	Provide feedback on current behaviour (BM3) Provide normative information about others’ behaviour and experiences (BM5) Facilitate relapse prevention and coping (BS2) Prompt self-recording (BS6) Provide information on withdrawal symptoms (RC6) Provide reassurance (RC10)	Face-to-face	Face-to-face
Session 4, week 7 Breaking habits	Provide feedback on current behaviour (BM3) Provide normative information about others’ behaviour and experiences (BM5) Facilitate barrier identification and problem solving (BS1) Facilitate relapse prevention and coping (BS2) Advise on changing routine (BS7) Advise on conserving mental resources (BS10) Advise on avoiding social cues for smoking (BS11) Advise on/facilitate use of social support (A2) Provide reassurance (RC10)	Face-to-face	Online
Session 5, week 9 Dealing with triggers	Provide rewards contingent on effort or progress (BM7) Facilitate relapse prevention and coping (BS2)	Face-to-face	Face-to-face
Session 6, week 11 Food for thought	Provide feedback on current behaviour (BM3) Offer/direct towards appropriate written materials (RC5) Elicit client views (RC8)	Face-to-face	Online
Session 7, week 14 Think differently	Provide feedback on current behaviour (BM3) Measure CO (BM11) Facilitate barrier identification and problem solving (BS1) Facilitate relapse prevention and coping (BS2) Prompt self-recording (BS6) Build general rapport (RC1) Elicit and answer questions (RC2)	Face-to-face	Face-to-face
Session 8, week 18 Do differently	Provide feedback on current behaviour (BM3) Facilitate barrier identification and problem solving (BS1) Facilitate relapse prevention and coping (BS2) Prompt self-recording (BS6) Tailor interactions appropriately (RD1) Build general rapport (RC1)	Face-to-face	Online
Session 9, week 22 Action plan	Provide feedback on current behaviour (BM3) Measure CO (BM11) Facilitate action planning/develop treatment plan (BS3) Build general rapport (RC1) Elicit client views (RC8)	Face-to-face	Face-to-face
Session 10, week 26 Closure	Provide feedback on current behaviour (BM3) Provide rewards contingent on successfully stopping smoking (BM4) Strengthen ex-smoker identity (BM8) Facilitate barrier identification and problem solving (BS1) Facilitate relapse prevention and coping (BS2) Facilitate goal setting (BS4) Set graded tasks (BS9) Advise on/facilitate use of social support (A2) Build general rapport (RC1) Offer/direct towards appropriate written materials (RC5) Elicit client views (RC8)	Face-to-face	Online

Codes: BM = Specific focus on behaviour (B) and addressing motivation (M); BS = Specific focus on behaviour (B) and maximising self-regulatory capacity/skills (S); A = Promote adjuvant activities (A); RC = General aspects of the interaction (R) focusing on general communication (C); RD = General aspects of the interaction (R) focusing on delivery of the intervention (D)

TAU is personalized to the patients’ needs and contains flexibility in quitting strategies. To allow for comparability this flexibility is also integrated in BSCT. At treatment start the patients will be asked to favor one of three quitting strategies:

1. Stop at once: the patient sets a quit date, makes a preparation plan and stops abruptly on the quit date.
2. Gradual change: the patient selects daily activities and contexts in which smoking is habitual and step-by-step continues these activities smoke free (for example when reading newspaper, Facebook, reading email, drinking coffee); finally, the patient sets a quit date. Being already accustomed to a range of daily habits without smoking will make it easier for the patient not to relapse.
3. Scheduled reduced smoking [21]: the patient gradually decreases the number of cigarettes at regular intervals; at the start the patient does not smoke less but becomes used to a fixed schedule, and in subsequent phases the number of cigarettes will be gradually reduced (100% → 75%; 75% → 50%; 50% → 25%) until the patient is ready to stop completely. This strategy systematically deconditions the cues. Although recent studies [22, 23] suggest that gradual cessation strategies – such as scheduled reduced smoking - may be sub-optimal compared to abrupt cessation, gradual cessation is still superior to non-treatment [21]. Offering scheduled reduced smoking broadens the target group for the cessation clinic, as it also allows patients who are initially reluctant to quit abruptly to enroll. Further, as it is an established part of TAU in this clinical setting, gradual cessation needs to be included in BSCT as well.

Both BSCT and TAU will consist of ten sessions with similar content spread over 6 months, with the frequency of sessions fading over time (six sessions within the first 3 months, four sessions within the final 3 months). Although participants may choose their preferred quitting strategy, this only marginally affects the content of the actual treatment that is delivered. Regardless of quitting strategy, the number and order of sessions is identical, as well as the effective components: the behavioral change techniques applied within sessions do not vary systematically. However, within the early sessions some differences may occur on a more detailed level within the BCTs (e.g. the timing of goal achievement within goal setting), due to quitting strategy.

All TAU sessions will take place at the SRP clinic while BSCT sessions will take place alternately face-to-face at the SRP (five sessions) and online (five sessions). This blended protocol resulted from a user centered design approach in which experts and counselors were involved in developing the most suitable mix of both delivery modes. The 50–50% blend of face-to-face and web-based sessions results in a considerable substitution by web-based interaction, while at the same time maintaining the intensity of the full intervention.

As in TAU, BSCT consists of both counselor-dependent and counselor-independent components. The counselor-dependent web-based components of BSCT are interactive and rely on (asynchronous) communication between counselor and patient. The counselor-independent components such as psycho-educational content or the smoking diary are used by the patients on their own and in their own time. In TAU these components are provided in a paper manual that clients take home. In BSCT, these components are accessible online. As such, both treatments are equivalent with regard to both content and intensity. An additional benefit of BSCT, though, is that the content of previous counselor-dependent components remains accessible as email correspondence saved online.

Both BSCT and TAU will cover the majority of the behavior change techniques that are used within individual behavioral support for smoking cessation [24], including those techniques that have shown to be reliably associated with better quit outcomes [25]. The distribution of the main behavior change techniques and the distribution of the face-to-face and web-based session are shown in Table 1.

### Measurements

The time-points of the follow-up measurements are tied to the estimated stop-date, which is appropriate for aid-to-cessation trials [26]. The expected stop-date is 3 months after treatment start, which is later than in common cessation treatments where quitting usually is expected within 1 to 3 weeks. In total there will be four follow-up measurements with measurement 3 and 4 (9 and 15 months follow-up) to be conducted at standard time points (i.e. 6 and 12 months after the expected stop-date):

1. 3 months after treatment start, expected stop-date, (3 months follow-up);
2. 6 months after treatment start, end of treatment and 3 months after expected stop-date, (6 months follow-up);
3. 9 months after treatment start, 3 months after end of treatment, 6 months after expected stop-date, (9 months follow-up); and
4. 15 months after treatment start, 9 months after end of treatment, 12 months after expected stop-date, (15 months follow-up).

A measurement schedule can be found in Table 2. The biochemical measurements will be done when the patient is at the hospital for a face-to-face session in week 1 (Exhaled CO; baseline/month 0), week 14 (Exhaled CO & Cotinine level; month 3) and week 22 (Exhaled CO; month 5). For the final biochemical measurement (Exhaled CO & Cotinine Level; month 15) which will take place 12 month after the expected stop date in month 3, the patient will have to return to the hospital. All other assessments will be done using online questionnaires which the patients will complete at home.

**Table 2:** Measurement schedule

Variables	Measurement at month					
	0	3	5	6	9	15
Primary outcome						
Cotinine level		X				X
Secondary outcomes						
Nicotine dependence (Fagerström)	X			X	X	
MAP-HSS + smoking related complaints of smokers	X			X	X	X
Depression, anxiety and stress (DASS21)	X			X	X	X
Quality of Life (Euroqol 5D)	X			X		
Smoking status	X	X		X	X	X
Adherence						X
Costs						X
Baseline predictors and moderators of treatment effect						
Internet Skills	X					
Readiness to change	X					
Attitude	X			X	X	
Social Influence	X					
Self-Efficacy	X			X		
Alcohol/substance (mis)use	X			X		
Descriptive variables						
Patient characteristics and medical history	X					
Smoking history	X					
Stop Smoking History	X					
Other information of interest						
Evaluation of treatment		X		X	X	X
Exhaled carbon monoxide (CO) level	X	X	X			X

The CO measurements in the study will serve as a backup for the cotinine measurements. They will only be analyzed in case the saliva samples are not usable. This backup strategy has been chosen because CO measurements are part of the routine stop-smoking treatment. To keep the burden for the participating patients as low as possible the measurements are linked to the scheduled face-to-face sessions. As the last face-to-face session in both treatment groups usually takes place in week 22 this moment has been chosen for the measurement to prevent the patient from travel time/work time loss for an extra appointment because of the study.

During the informed consent procedure all participants will receive a patient information letter that outlines the burden of participation, including the online questionnaires. By stressing the importance of the online questionnaires we try to increase patient commitment. During the trial, completion of the questionnaires will be checked after 2 weeks. Participants not completing the questionnaire will receive four weekly reminders: first twice via email, then twice by telephone. If a participant signals to be struggling with completing the questionnaire online, we will offer a paper version of the questionnaire, which will be sent including a return envelope.

## Instruments

### Primary outcome (Cotinine level)

The primary outcome parameter will be biochemically validated prolonged abstinence [26] at 15 months from the start of the smoking cessation treatment. Saliva cotinine level will be measured as biochemical verification of abstinence only by those patients that report quitting smoking in the previous online questionnaires [27]. Prolonged abstinence is defined as having salivary cotinine levels < 20 ng/ml [28] that validate both the self- reported abstinence after the self-chosen stop date – usually 3 months after start - and the self-reported abstinence at 15 month follow-up. Patients not reporting abstinence or with a higher cotinine level on any of these follow-ups will be regarded as smokers as well as patients who are lost to follow-up. A 0,5–1 ml salivary sample will be collected for cotinine assessment by means of a Salivette (Sarstedt AG & Co., Nümbrecht, Germany). Under supervision, patients will have to chew on a cotton swab for 1min to stimulate the saliva flow rate. All saliva specimens will be frozen until assayed and transported to the laboratory for the determination of the cotinine level using a gas chromatography technique.

### Secondary outcomes

#### Nicotine dependence

Fagerström Test for Nicotine Dependence (FTND) [29] is the most commonly used tool for the assessment of nicotine dependency. The scores obtained on the test permit the classification of nicotine dependence into five levels: very low (0 to 2 points); low (3 to 4 points); moderate (five points); high (6 to 7 points); and very high (8 to 10 points). The instrument evaluates for example time from awakening to the day’s first cigarette, smoking when bed-ridden with illness, and difficulty in refraining from smoking when prohibited.

### MAP-HSS + smoking related complaints of smokers

The MAP-HSS is a ten-item health scale, which was adapted from the Opiate Treatment Index [30]. Each item is scored on a five-point Likert-type scale, ranging from 0 (complaint never present in the previous 30 days) to 4 (complaint always present in the previous 30 days), resulting in a total scale-score ranging from 0 to 40. In addition to MAP-HSS the patients will be asked to scale 16 typical smoking related complaints (for example cold hands and feet, cough, pale skin, pain in the lung). An overall score of physical complaints will be calculated by adding MAP-HSS and the additional smoking related complaints.

### Depression, anxiety and stress (DASS-21)

The DASS21 [31, 32] is a consistent, valid and reliable set of three self-report scales designed to measure the negative emotional states of depression, anxiety and stress. Each of the three DASS scales contains seven items. Patients will be asked to use 4-point severity/frequency scales to rate the extent to which they have experienced each state over the past week. Scores for Depression, Anxiety and Stress will be calculated by summing the scores for the relevant items.

### Quality of life (Euroqol 5D)

The EuroQol-5D [33] is a generic quality-of-life (QoL) instrument which consists of five domains: mobility, self-care, usual activities, pain/discomfort and anxiety/depression. There are three response alternatives for each domain. The EQ-5D index is obtained by means of applying predetermined weights to the five domains. The EQ-5D index is a societal-based numerical quantification of the patients’ health status which can range from 0 (death) to 1 (perfect health status). In addition to the five domains, EuroQol-5D also offers an overall rating for quality of life by means of a visual analogue scale (VAS). The VAS is a vertical line from worst (0) to best state of health (100).

### Smoking status

Smoking status comprises self-reported smoking related variables such as quit attempts (>24 h), number of relapses, or the amount of daily tobacco consumption (cigarettes, self-rolled cigarettes, cigarillos, e-cigarettes). All smoking status variables are based on a standardized questionnaire for Dutch tobacco research [34].

### Adherence

In order to find out how the web-based application is used, real-time logdata will be collected to track individual use. These log files will be used for identifying user profiles and to gain insight into adherence to the application, usage patterns that emerge, and what elements of the application are used. This information will provide insight in how the application (both content and system) matches with its users. In addition, information about the type and number of BCTs taken from the patient’s record, which is kept by the counsellor, will be analysed to calculate the level of adherence.

### Costs

All direct treatment-related costs generated by the care providers and the patients: Costs will be calculated based on hours spent by the counsellors (including for no shows), patients’ travel costs, and maintenance of the web-based infrastructure.

### Baseline predictors and moderators of treatment effect

#### Internet skills

Internet skills will be measured using an online questionnaire based on conceptual definitions for internet skills [17]. This conceptual definition includes two major skill areas (medium-related Internet skills and content-related Internet skills), which contain in total five minor skill areas. Medium-related Internet skills include operational (for example operating an Internet browser or a search engine) and formal (for example maintaining a sense of location when on the Internet) skills. Content-related Internet skills include informational (for example defining search options or queries), communication (for example searching, selecting, reaching and evaluating contacts online) and strategic (for example taking advantage of the Internet) skills. A ten item questionnaire [35] will be used to measure internet skills with a 5-point Likert scale, resulting in a score range from 10 (unskilled) to 50 (highly skilled).

### Readiness to change

Readiness to change will be measured using the algorithm to detect the stage of change in smokers [36]. The expected stop moment (within 1 month versus within 2 or 3 months) offers the possibility to distinguish between the contemplation and preparation stage of change.

### Attitude, social influence and self-efficacy

According to the ASE Model [37, 38] the intention to stop smoking is determined by three motivational factors: Attitude, Social Influence and Self-Efficacy. Attitude [39, 40] refers to the overall evaluation of smoking cessation. Attitude will be measured with an indirect, belief-based scale for perceived pros (four items: improved health for the patient, improved health for the patient’s personal environment, lower risk of lung cancer, improved self-satisfaction) and cons (four items: suffering withdrawal symptoms, missing smoking, less ability to relax, feeling bored). Social influence refers to three distinctive constructs: social norms, perceived behaviour of others and direct support. It will be measured recording if the patient is stimulated to stop smoking by acquaintances, if his/her partners is a smoker and how many of his/her acquaintances are smokers. Self-Efficacy [41] refers to the confidence in the ability to refrain from smoking in specific high-risk situations, i.e. the situations in which the quitter is tempted to relapse. It will be measured recording six typical relapse situations (e.g. stress or party). The three constructs of the ASE model will be measured using standardized questions developed by the former Dutch foundation STIVORO [34, 42].

### Alcohol and substance (mis)use

Alcohol (mis)use will be measured using the Five-shot questionnaire on heavy drinking [43]. Only if a patient declares that he/she is consuming alcohol/substances at all, additional questions will be asked to keep the burden for the patient as low as possible. The additional questions will record the frequency and amount of alcohol consumption, feelings of anger and guilt related to drinking, and if the patient drinks in the morning to cope with hangover. Substance (mis)use will be measured asking for (recreational-)drug use in general. If the patient declares to use (recreational-)drugs additional questions will ask for type of (recreational-)drug and frequency and duration of use.

### Descriptive variables

#### Patient characteristics and medical history

Demographical data (sex, age, nationality, cultural background, marital status, children, housing, education, source of income, main activity) will be collected using an online questionnaire. Medical history will be recorded from medical charts.

### Smoking history

Smoking history will be measured using an online questionnaire from the longitudinal Vlagtwedde-Vlaardingen Study (1965 to 1990) [44] recording the age of first smoking attempts and the numbers of years and number of cigarettes/day that the patient was smoking in each decade.

### Stop smoking history

Earlier attempts to stop smoking will be recorded using an online questionnaire asking: if there were earlier stop smoking attempts; when the last stop smoking attempt was; how long the non-smoking phase was; and when the last stop smoking attempt was, which was successful for more than 24 h.

### Other information of interest

#### Evaluation of treatment

Three month after start of the treatment, at the end of the treatment (6 month after start) and during the follow-up measurements (9 and 15 month after start) patients will be asked to report their experiences with the different aspects of the treatment program. Patients can rate satisfaction with the program by grading all separate types of contact, assessing the overall contact with their counsellors, and reporting their own perception of improvements. In addition, they will be asked to report on adherence, results and benefits, gained insights, the use of co- interventions, and the use of NRT. Furthermore, they will be asked for improvement suggestions.

#### Exhaled carbon monoxide (CO) level

The measurement of exhaled carbon monoxide (CO) level provides an immediate, non-invasive method of assessing smoking status [45]. A breath CO level of 5 ppm is taken as the cut-off between smokers and non-smokers (5 ppm or higher = smoker, less than 5 ppm = non-smoker). Breath CO monitoring will be performed using a piCo Smokerlyzer (Bedfont Instruments: Kent, UK), a portable CO monitor.

The level of exhaled carbon monoxide (CO) level will be recorded because the CO measurement is already part of the treatment so that these data is easily available. Because the validation of the smoking cessation is done with cotinine tests (see above) the CO data will only be used if cotinine measurements are missing and to provide data for future research such as for example comparing different measurement techniques.

### Sample size

For this RCT 344 patients will be needed. This is based on the following assumptions and calculations.

Since we expect that BSCT will be at least non-inferior to TAU concerning prolonged abstinence, we conduct a non-inferiority trial. Furthermore, we expect BSCT to be better at secondary factors such as costs, adherence and satisfaction [46].

Based on previous studies involving smoking cessation treatment within the organization involved in this RCT [4, 5] and meta analyses [3], a cotinine-validated prolonged 12 months’ abstinence rate of 10% (based on intention-to-treat analysis) with TAU is expected. Based on the expected benefits of BSCT the estimated abstinence rate for BSCT is 15%. If BSCT leads to an abstinence rate of not lower than 5% it will be regarded as non-inferior. With a power of 80% and α of 0,025 172 patients per group are needed for this RCT (calculated with PASS).

The 5% criterion is based on the three assumptions described below:

1. A validated prolonged abstinence rate of 5% may still be considered as superior to (1) a non-intervention condition which is estimated at a 1.4% abstinence rate and to (2) a 2.6% abstinence in a minimal intervention condition in clinical populations such as COPD patients [47].
2. In a worst-case scenario, BSCT patients will fail to use the web-based part of the intervention completely, and adhere to the face-to-face component only. This would reduce their exposure to the intervention by 50% compared to full adherence to TAU. Assuming a linear dose–response relationship of intervention intensity and likelihood of abstinence the 10% abstinence rate estimated for TAU would then be reduced by 50% to a 5% abstinence rate.
3. Although a 5% abstinence rate is considerably lower than the estimated 10% in TAU, the secondary benefits of BSCT - such as client satisfaction - need to be taken into account. Thus, even at a lower effectiveness we expect that BSCT can still be the preferred treatment.

Based on the experience that approximately 360 patients per year start a cessation treatment at SRP, it is expected that recruitment, treatment and follow-measurements of the 344 patients needed for this RCT will take 3 to 4 years.

#### Handling of study dropouts

If a subject is prematurely withdrawn or withdraws from participation in the study for whatever reason, the statistical analysis will be conducted following the intention-to-treat principle [48], assuming that missing cases are at their baseline level. This will produce conservative estimates of smoking abstinence but will still allow for treatment outcomes that are based on the entire sample. Patients who fail to keep an appointment will be contacted and if possible, will be rescheduled for another appointment, ideally within 7 days of the missed appointment.

### Data management

The handling of personal data will comply with the Personal Data Protection Act in The Netherlands. Data will be recorded using the two ways of data collection described below.

1. Data from the face-to-face contacts will be recorded on data collection forms and centrally collected at Medisch Spectrum Twente. The study data manager will record all the collected data in a Microsoft Access 2007 database.
2. The majority of data will be recorded by Tactus Addiction Treatment – a regional addiction care organization with experience in web-based treatment - using online questionnaires which will be offered to both treatment groups. Individual patients and counsellors will have a login with username and password secured by Secure Sockets Layer to the application. All data transferred between the patient’s personal computer and the application will be encrypted and sent via the https protocol. All data will be encrypted and stored on servers in secure data centres within the Netherlands. Daily backups of the server will be made to ensure further data security.

### Data analysis/Statistical methods

Baseline characteristics will be displayed as means with standard deviations (SD) or medians with interquartile range (IQR) for continuous variables depending on the distribution of the variable; categorical variables will be displayed as counts with corresponding percentages. Differences between the two treatment groups in terms of continuous variables will be tested by the independent *T*-test or the Wilcoxon rank sum test, depending on the distribution of the variable. Differences in categorical variables will be tested by the Chi-square test or the Fisher exact test.

The non-inferiority between BSCT and TAU in salivary cotinine validated 12 months’ prolonged abstinence rate will be analyzed by calculating the 95% confidence interval of the observed difference in the abstinence rate and by comparing that to the previously defined non-inferiority margin of 5%.

To assess whether BSCT leads to a decrease in treatment costs compared to TAU, an incremental cost-effectiveness ratio (ICER) will be calculated using treatment costs and abstinence rate.

To assess whether BSCT leads to improved satisfaction among patients and counsellors compared to TAU, satisfaction (based on the middle and long-term evaluation) will be tested between the two groups by applying the independent *T*-test or the Wilcoxon rank sum test.

Both baseline predictors - moderators of intervention effect - and dynamic predictors - mechanisms through which effects occur - will be tested using moderator and mediator analyses [49, 50], including all potential co-variates such as nicotine dependence, cognitive determinants (for example attitude and self-efficacy), medical conditions and mental states (for example depression and anxiety), adherence and internet skills.

Whether the level of adherence to BSCT is related to prolonged abstinence will be tested by the independent *T*-test or the Wilcoxon rank sum test.

All analyses will be performed based on the intention-to-treat principle and will be performed in SPSS version 20.0.

## Discussion

To the best of our knowledge, this RCT will be the first study to examine the effectiveness of a blended smoking cessation treatment compared to purely face-to-face treatment. It will also be the first study to explore patient satisfaction, adherence, cost-effectiveness, and active ingredients of a blended smoking cessation treatment. The main three strengths of the LiveSmokefree Study are: (1) The blended smoking cessation treatment explored in this study was developed by a team in which all relevant stakeholder actively participated; (2) it demonstrates high ecological validity involving the heterogeneous population of regular patients of an outpatient smoking cessation clinic; and (3) it includes a long term biochemically validated follow-up assessment. Main objective is to test whether face-to-face-smoking cessation treatment can be substituted by a less demanding and more patient-friendly blended treatment with similar outcomes. As our clinical experience with blended smoking cessation treatment evolves, this trial will contribute to the understanding of the influence of blended treatment on both the stop smoking process and the patient’s experience. The conduct of this trial and its findings will substantially strengthen the evidence base regarding new delivery modes of smoking cessation treatment. If blended smoking cessation treatment is shown to be non-inferior on effectiveness while offering secondary benefits, then dissemination to clinical practice should be warranted. Such secondary benefits may be lower treatment costs and higher user friendliness.

However, there are also limitations in this study. First, by allowing participants in both arms to opt for one of three different quitting strategies (stop at once, gradual change, scheduled reduced smoking), heterogeneity is introduced within the treatment. As allocation to the treatment groups is stratified on this criterion, however, all three quit strategies will be distributed equally over both groups and therefore not affect the internal validity of this trial. As a result, the main objective of this trial, i.e. comparing two modes of delivery of a treatment with identical content and intensity, is safeguarded. External validity may be somewhat impeded, though, as most cessation interventions in previous trials do not display such a flexible, preference-based, quit approach. To explore this issue sub-analyses of program effects among participants within each quit strategy will be considered, depending on sufficient sample size within subgroups. Alternately, moderation by quit strategies or by preference-based personalization requires testing in a more complex trial design with multiple study arms in future research.

Despite this obvious limitation, the personalized quitting strategy can also be considered a strength of the treatment, as it broadens the treatment to a larger target group and increases the potential reach among smokers.

Another limitation that will merit discussion of the practical relevance is the rather inflexible approach of blending in the treatment (five web-based sessions and five face-to-face sessions in a fixed sequence and with equivalent content) that is used to allow for comparability. This inflexible approach may limit the potential of blending. Blending web-based and face-to-face intervention could - in extremis - lead to a flexible exchangeability of all intervention components, which would foster a treatment that is highly tailored to the patient’s needs and abilities. This would allow both the counsellor and the patient to choose a preferred blend of web-based or face-to-face delivery, while maintaining efficacy. Future studies should explore such benefits of flexibly blended treatment options.

### Current status

Recruitment of patients started in May 2015.

## Abbreviations

BSCT: Blended smoking cessation treatment
FtF: face-to-face
GP: General practitioner
ICER: Incremental cost-effectiveness ratio
IQR: Interquartile range
MREC: Medical Research Ethics Committee
MST: Medisch Spectrum Twente hospital
QoL: Quality of life
RCT: Randomized controlled trial
SD: Standard deviation
SRP: Outpatient Smoking Cessation Clinic of Medisch Spetrum Twente (Dutch: Stoppen-met-Roken-Poli)
TAU: Treatment as usual
VAS: Visual analog scale
